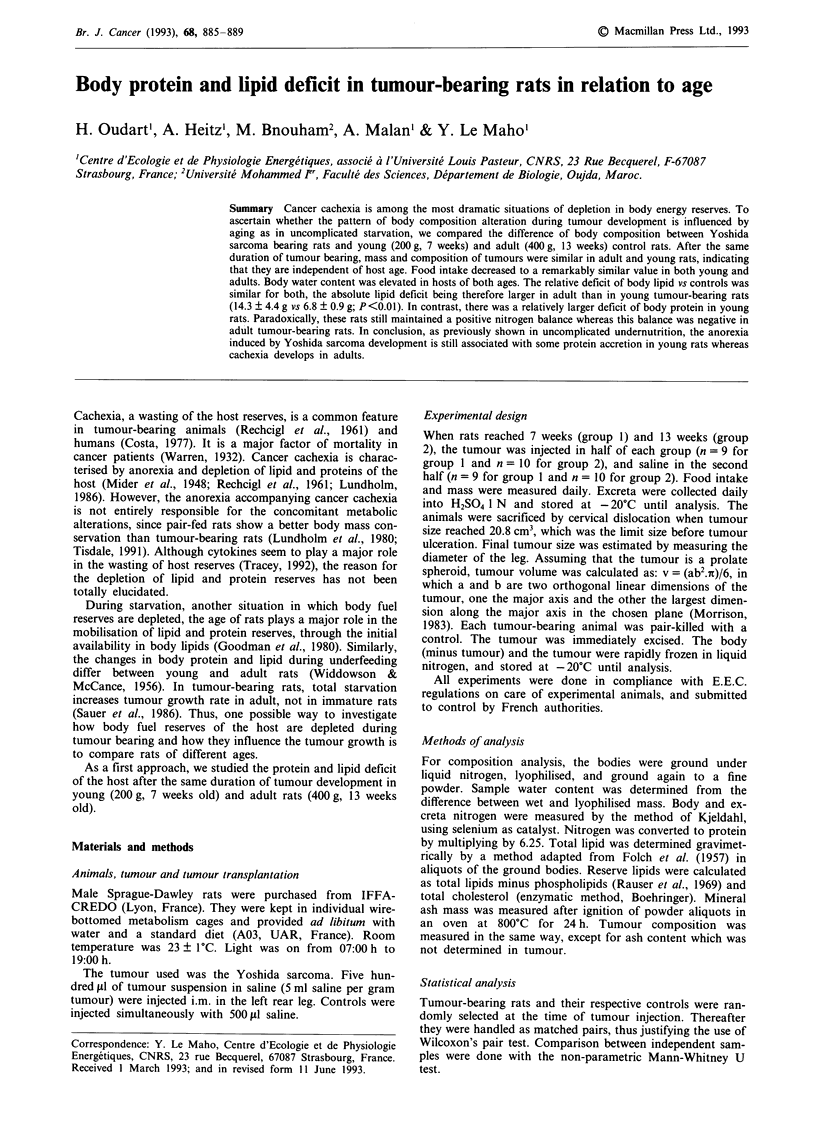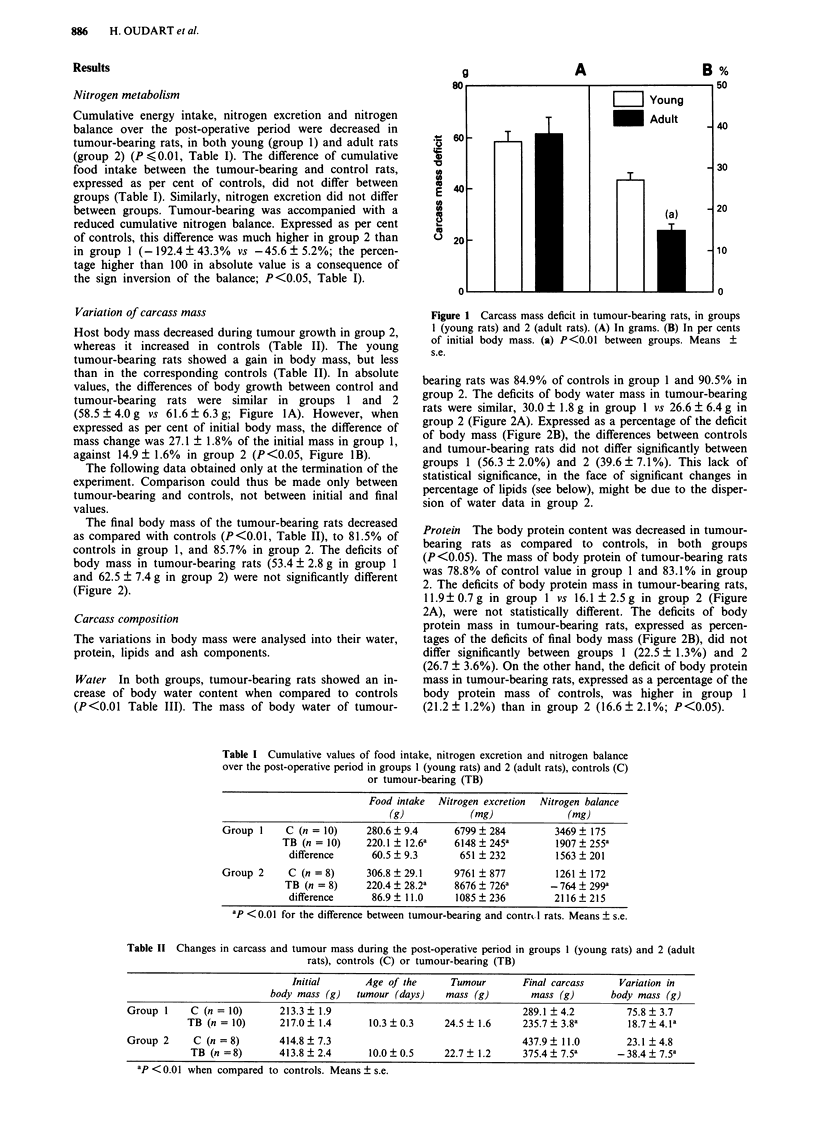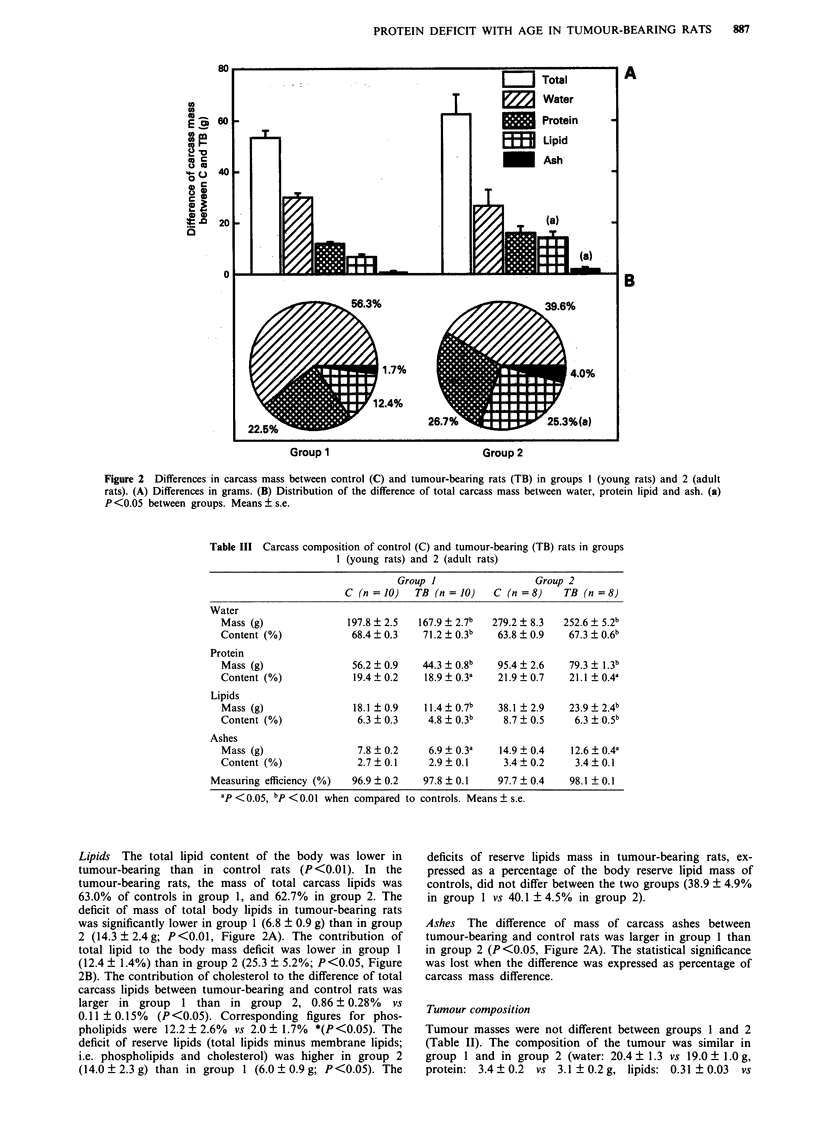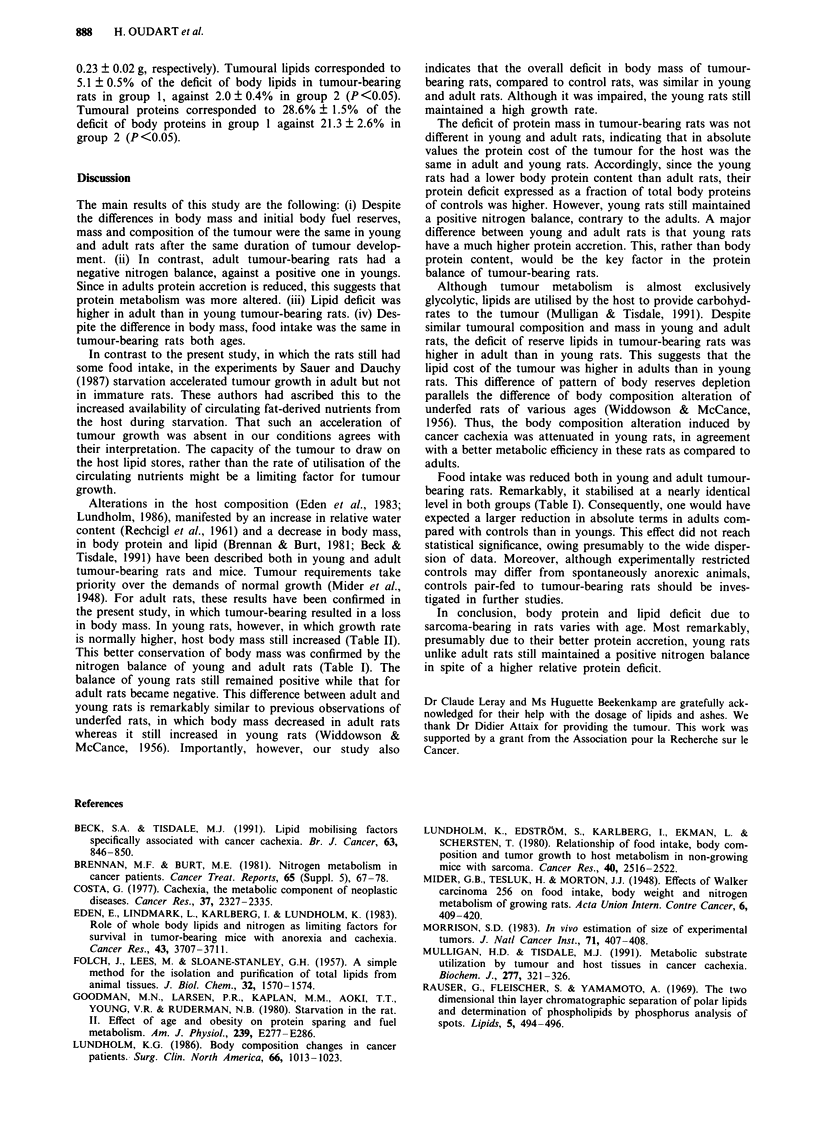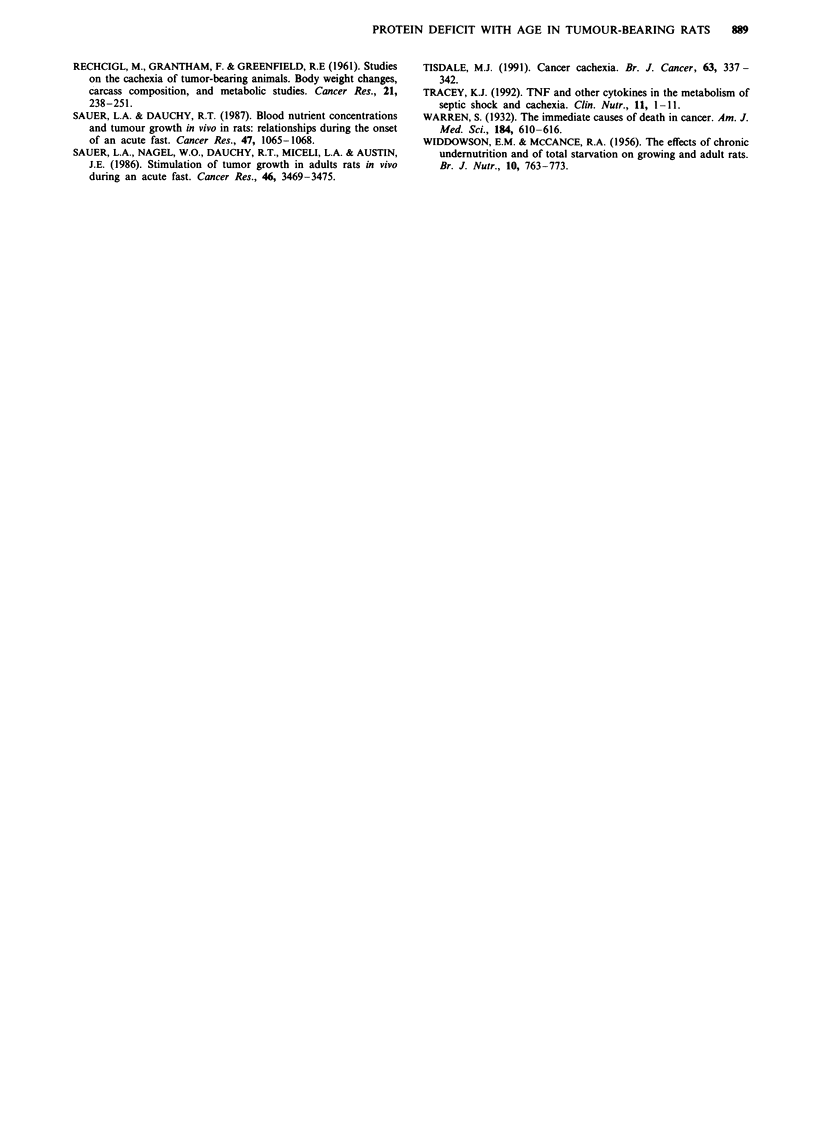# Body protein and lipid deficit in tumour-bearing rats in relation to age.

**DOI:** 10.1038/bjc.1993.450

**Published:** 1993-11

**Authors:** H. Oudart, A. Heitz, M. Bnouham, A. Malan, Y. Le Maho

**Affiliations:** Centre d'Ecologie et de Physiologie Energétiques, CNRS, Strasbourg, France.

## Abstract

Cancer cachexia is among the most dramatic situations of depletion in body energy reserves. To ascertain whether the pattern of body composition alteration during tumour development is influenced by aging as in uncomplicated starvation, we compared the difference of body composition between Yoshida sarcoma bearing rats and young (200 g, 7 weeks) and adult (400 g, 13 weeks) control rats. After the same duration of tumour bearing, mass and composition of tumours were similar in adult and young rats, indicating that they are independent of host age. Food intake decreased to a remarkably similar value in both young and adults. Body water content was elevated in hosts of both ages. The relative deficit of body lipid vs controls was similar for both, the absolute lipid deficit being therefore larger in adult than in young tumour-bearing rats (14.3 +/- 4.4 g vs 6.8 +/- 0.9 g; P < 0.01). In contrast, there was a relatively larger deficit of body protein in young rats. Paradoxically, these rats still maintained a positive nitrogen balance whereas this balance was negative in adult tumour-bearing rats. In conclusion, as previously shown in uncomplicated undernutrition, the anorexia induced by Yoshida sarcoma development is still associated with some protein accretion in young rats whereas cachexia develops in adults.


Br. J. Cancer (1993), 68, 885-889 © Macmillan Press Ltd., 1993

### Body protein and lipid deficit in tumour-bearing rats in relation to age

H. Oudart[1], A. Heitz[1], M. Bnouham[2], A. Malan[1] & Y. Le Maho[1]

[1]*Centre d'Ecologie et de Physiologie Energétiques, associé à l'Université Louis Pasteur, CNRS, 23 Rue Becquerel, F-67087 Strasbourg, France;* [2]*Université Mohammed I[er], Faculté des Sciences, Département de Biologie, Oujda, Maroc.*

**Summary** Cancer cachexia is among the most dramatic situations of depletion in body energy reserves. To ascertain whether the pattern of body composition alteration during tumour development is influenced by aging as in uncomplicated starvation, we compared the difference of body composition between Yoshida sarcoma bearing rats and young (200 g, 7 weeks) and adult (400 g, 13 weeks) control rats. After the same duration of tumour bearing, mass and composition of tumours were similar in adult and young rats, indicating that they are independent of host age. Food intake decreased to a remarkably similar value in both young and adults. Body water content was elevated in hosts of both ages. The relative deficit of body lipid *vs* controls was similar for both, the absolute lipid deficit being therefore larger in adult than in young tumour-bearing rats ($14.3 \pm 4.4$ g *vs* $6.8 \pm 0.9$ g; $P<0.01$). In contrast, there was a relatively larger deficit of body protein in young rats. Paradoxically, these rats still maintained a positive nitrogen balance whereas this balance was negative in adult tumour-bearing rats. In conclusion, as previously shown in uncomplicated undernutrition, the anorexia induced by Yoshida sarcoma development is still associated with some protein accretion in young rats whereas cachexia develops in adults.

Cachexia, a wasting of the host reserves, is a common feature in tumour-bearing animals (Rechcigl *et al.*, 1961) and humans (Costa, 1977). It is a major factor of mortality in cancer patients (Warren, 1932). Cancer cachexia is characterised by anorexia and depletion of lipid and proteins of the host (Mider *et al.*, 1948; Rechcigl *et al.*, 1961; Lundholm, 1986). However, the anorexia accompanying cancer cachexia is not entirely responsible for the concomitant metabolic alterations, since pair-fed rats show a better body mass conservation than tumour-bearing rats (Lundholm *et al.*, 1980; Tisdale, 1991). Although cytokines seem to play a major role in the wasting of host reserves (Tracey, 1992), the reason for the depletion of lipid and protein reserves has not been totally elucidated.

During starvation, another situation in which body fuel reserves are depleted, the age of rats plays a major role in the mobilisation of lipid and protein reserves, through the initial availability in body lipids (Goodman *et al.*, 1980). Similarly, the changes in body protein and lipid during underfeeding differ between young and adult rats (Widdowson & McCance, 1956). In tumour-bearing rats, total starvation increases tumour growth rate in adult, not in immature rats (Sauer *et al.*, 1986). Thus, one possible way to investigate how body fuel reserves of the host are depleted during tumour bearing and how they influence the tumour growth is to compare rats of different ages.

As a first approach, we studied the protein and lipid deficit of the host after the same duration of tumour development in young (200 g, 7 weeks old) and adult rats (400 g, 13 weeks old).

### Materials and methods

### *Animals, tumour and tumour transplantation*

Male Sprague-Dawley rats were purchased from IFFA-CREDO (Lyon, France). They were kept in individual wire-bottomed metabolism cages and provided *ad libitum* with water and a standard diet (A03, UAR, France). Room temperature was $23 \pm 1$°C. Light was on from 07:00 h to 19:00 h.

The tumour used was the Yoshida sarcoma. Five hundred µl of tumour suspension in saline (5 ml saline per gram tumour) were injected i.m. in the left rear leg. Controls were injected simultaneously with 500 µl saline.

Correspondence: Y. Le Maho, Centre d'Ecologie et de Physiologie Energétiques, CNRS, 23 rue Becquerel, 67087 Strasbourg, France.
Received 1 March 1993; and in revised form 11 June 1993.

### *Experimental design*

When rats reached 7 weeks (group 1) and 13 weeks (group 2), the tumour was injected in half of each group ($n = 9$ for group 1 and $n = 10$ for group 2), and saline in the second half ($n = 9$ for group 1 and $n = 10$ for group 2). Food intake and mass were measured daily. Excreta were collected daily into $H_2SO_4$ 1 N and stored at $-20$°C until analysis. The animals were sacrificed by cervical dislocation when tumour size reached 20.8 $cm^3$, which was the limit size before tumour ulceration. Final tumour size was estimated by measuring the diameter of the leg. Assuming that the tumour is a prolate spheroid, tumour volume was calculated as: $v = (ab^2.\pi)/6$, in which a and b are two orthogonal linear dimensions of the tumour, one the major axis and the other the largest dimension along the major axis in the chosen plane (Morrison, 1983). Each tumour-bearing animal was pair-killed with a control. The tumour was immediately excised. The body (minus tumour) and the tumour were rapidly frozen in liquid nitrogen, and stored at $-20$°C until analysis.

All experiments were done in compliance with E.E.C. regulations on care of experimental animals, and submitted to control by French authorities.

### *Methods of analysis*

For composition analysis, the bodies were ground under liquid nitrogen, lyophilised, and ground again to a fine powder. Sample water content was determined from the difference between wet and lyophilised mass. Body and excreta nitrogen were measured by the method of Kjeldahl, using selenium as catalyst. Nitrogen was converted to protein by multiplying by 6.25. Total lipid was determined gravimetrically by a method adapted from Folch *et al.* (1957) in aliquots of the ground bodies. Reserve lipids were calculated as total lipids minus phospholipids (Rauser *et al.*, 1969) and total cholesterol (enzymatic method, Boehringer). Mineral ash mass was measured after ignition of powder aliquots in an oven at 800°C for 24 h. Tumour composition was measured in the same way, except for ash content which was not determined in tumour.

### *Statistical analysis*

Tumour-bearing rats and their respective controls were randomly selected at the time of tumour injection. Thereafter they were handled as matched pairs, thus justifying the use of Wilcoxon's pair test. Comparison between independent samples were done with the non-parametric Mann-Whitney U test.

### Results

### Nitrogen metabolism

Cumulative energy intake, nitrogen excretion and nitrogen balance over the post-operative period were decreased in tumour-bearing rats, in both young (group 1) and adult rats (group 2) ($P \leqslant 0.01$, Table I). The difference of cumulative food intake between the tumour-bearing and control rats, expressed as per cent of controls, did not differ between groups (Table I). Similarly, nitrogen excretion did not differ between groups. Tumour-bearing was accompanied with a reduced cumulative nitrogen balance. Expressed as per cent of controls, this difference was much higher in group 2 than in group 1 (−192.4 ± 43.3% *vs* −45.6 ± 5.2%; the percentage higher than 100 in absolute value is a consequence of the sign inversion of the balance; $P<0.05$, Table I).

### Variation of carcass mass

Host body mass decreased during tumour growth in group 2, whereas it increased in controls (Table II). The young tumour-bearing rats showed a gain in body mass, but less than in the corresponding controls (Table II). In absolute values, the differences of body growth between control and tumour-bearing rats were similar in groups 1 and 2 (58.5 ± 4.0 g *vs* 61.6 ± 6.3 g; Figure 1A). However, when expressed as per cent of initial body mass, the difference of mass change was 27.1 ± 1.8% of the initial mass in group 1, against 14.9 ± 1.6% in group 2 ($P<0.05$, Figure 1B).

The following data obtained only at the termination of the experiment. Comparison could thus be made only between tumour-bearing and controls, not between initial and final values.

The final body mass of the tumour-bearing rats decreased as compared with controls ($P<0.01$, Table II), to 81.5% of controls in group 1, and 85.7% in group 2. The deficits of body mass in tumour-bearing rats (53.4 ± 2.8 g in group 1 and 62.5 ± 7.4 g in group 2) were not significantly different (Figure 2).

### Carcass composition

The variations in body mass were analysed into their water, protein, lipids and ash components.

*Water* In both groups, tumour-bearing rats showed an increase of body water content when compared to controls ($P<0.01$ Table III). The mass of body water of tumour-bearing rats was 84.9% of controls in group 1 and 90.5% in group 2. The deficits of body water mass in tumour-bearing rats were similar, 30.0 ± 1.8 g in group 1 *vs* 26.6 ± 6.4 g in group 2 (Figure 2A). Expressed as a percentage of the deficit of body mass (Figure 2B), the differences between controls and tumour-bearing rats did not differ significantly between groups 1 (56.3 ± 2.0%) and 2 (39.6 ± 7.1%). This lack of statistical significance, in the face of significant changes in percentage of lipids (see below), might be due to the dispersion of water data in group 2.

**Figure 1** Carcass mass deficit in tumour-bearing rats, in groups 1 (young rats) and 2 (adult rats). (**A**) In grams. (**B**) In per cents of initial body mass. (**a**) $P<0.01$ between groups. Means ± s.e.

*Protein* The body protein content was decreased in tumour-bearing rats as compared to controls, in both groups ($P<0.05$). The mass of body protein of tumour-bearing rats was 78.8% of control value in group 1 and 83.1% in group 2. The deficits of body protein mass in tumour-bearing rats, 11.9 ± 0.7 g in group 1 *vs* 16.1 ± 2.5 g in group 2 (Figure 2A), were not statistically different. The deficits of body protein mass in tumour-bearing rats, expressed as percentages of the deficits of final body mass (Figure 2B), did not differ significantly between groups 1 (22.5 ± 1.3%) and 2 (26.7 ± 3.6%). On the other hand, the deficit of body protein mass in tumour-bearing rats, expressed as a percentage of the body protein mass of controls, was higher in group 1 (21.2 ± 1.2%) than in group 2 (16.6 ± 2.1%; $P<0.05$).

**Table I** Cumulative values of food intake, nitrogen excretion and nitrogen balance over the post-operative period in groups 1 (young rats) and 2 (adult rats), controls (C) or tumour-bearing (TB)

| | | *Food intake (g)* | *Nitrogen excretion (mg)* | *Nitrogen balance (mg)* |
|---|---|---|---|---|
| Group 1 | C ($n$ = 10) | 280.6 ± 9.4 | 6799 ± 284 | 3469 ± 175 |
| | TB ($n$ = 10) | 220.1 ± 12.6[a] | 6148 ± 245[a] | 1907 ± 255[a] |
| | difference | 60.5 ± 9.3 | 651 ± 232 | 1563 ± 201 |
| Group 2 | C ($n$ = 8) | 306.8 ± 29.1 | 9761 ± 877 | 1261 ± 172 |
| | TB ($n$ = 8) | 220.4 ± 28.2[a] | 8676 ± 726[a] | −764 ± 299[a] |
| | difference | 86.9 ± 11.0 | 1085 ± 236 | 2116 ± 215 |

[a]$P<0.01$ for the difference between tumour-bearing and control rats. Means ± s.e.

**Table II** Changes in carcass and tumour mass during the post-operative period in groups 1 (young rats) and 2 (adult rats), controls (C) or tumour-bearing (TB)

| | | *Initial body mass (g)* | *Age of the tumour (days)* | *Tumour mass (g)* | *Final carcass mass (g)* | *Variation in body mass (g)* |
|---|---|---|---|---|---|---|
| Group 1 | C ($n$ = 10) | 213.3 ± 1.9 | | | 289.1 ± 4.2 | 75.8 ± 3.7 |
| | TB ($n$ = 10) | 217.0 ± 1.4 | 10.3 ± 0.3 | 24.5 ± 1.6 | 235.7 ± 3.8[a] | 18.7 ± 4.1[a] |
| Group 2 | C ($n$ = 8) | 414.8 ± 7.3 | | | 437.9 ± 11.0 | 23.1 ± 4.8 |
| | TB ($n$ = 8) | 413.8 ± 2.4 | 10.0 ± 0.5 | 22.7 ± 1.2 | 375.4 ± 7.5[a] | −38.4 ± 7.5[a] |

[a]$P<0.01$ when compared to controls. Means ± s.e.

**Figure 2** Differences in carcass mass between control (C) and tumour-bearing rats (TB) in groups 1 (young rats) and 2 (adult rats). (A) Differences in grams. (B) Distribution of the difference of total carcass mass between water, protein lipid and ash. (a) $P<0.05$ between groups. Means ± s.e.

**Table III** Carcass composition of control (C) and tumour-bearing (TB) rats in groups 1 (young rats) and 2 (adult rats)

| | *Group 1* | | *Group 2* | |
|---|---|---|---|---|
| | *C (n = 10)* | *TB (n = 10)* | *C (n = 8)* | *TB (n = 8)* |
| Water | | | | |
| Mass (g) | 197.8 ± 2.5 | 167.9 ± 2.7[b] | 279.2 ± 8.3 | 252.6 ± 5.2[b] |
| Content (%) | 68.4 ± 0.3 | 71.2 ± 0.3[b] | 63.8 ± 0.9 | 67.3 ± 0.6[b] |
| Protein | | | | |
| Mass (g) | 56.2 ± 0.9 | 44.3 ± 0.8[b] | 95.4 ± 2.6 | 79.3 ± 1.3[b] |
| Content (%) | 19.4 ± 0.2 | 18.9 ± 0.3[a] | 21.9 ± 0.7 | 21.1 ± 0.4[a] |
| Lipids | | | | |
| Mass (g) | 18.1 ± 0.9 | 11.4 ± 0.7[b] | 38.1 ± 2.9 | 23.9 ± 2.4[b] |
| Content (%) | 6.3 ± 0.3 | 4.8 ± 0.3[b] | 8.7 ± 0.5 | 6.3 ± 0.5[b] |
| Ashes | | | | |
| Mass (g) | 7.8 ± 0.2 | 6.9 ± 0.3[a] | 14.9 ± 0.4 | 12.6 ± 0.4[a] |
| Content (%) | 2.7 ± 0.1 | 2.9 ± 0.1 | 3.4 ± 0.2 | 3.4 ± 0.1 |
| Measuring efficiency (%) | 96.9 ± 0.2 | 97.8 ± 0.1 | 97.7 ± 0.4 | 98.1 ± 0.1 |

[a]$P<0.05$, [b]$P<0.01$ when compared to controls. Means ± s.e.

*Lipids* The total lipid content of the body was lower in tumour-bearing than in control rats ($P<0.01$). In the tumour-bearing rats, the mass of total carcass lipids was 63.0% of controls in group 1, and 62.7% in group 2. The deficit of mass of total body lipids in tumour-bearing rats was significantly lower in group 1 (6.8 ± 0.9 g) than in group 2 (14.3 ± 2.4 g; $P<0.01$, Figure 2A). The contribution of total lipid to the body mass deficit was lower in group 1 (12.4 ± 1.4%) than in group 2 (25.3 ± 5.2%; $P<0.05$, Figure 2B). The contribution of cholesterol to the difference of total carcass lipids between tumour-bearing and control rats was larger in group 1 than in group 2, 0.86 ± 0.28% *vs* 0.11 ± 0.15% ($P<0.05$). Corresponding figures for phospholipids were 12.2 ± 2.6% *vs* 2.0 ± 1.7% *($P<0.05$). The deficit of reserve lipids (total lipids minus membrane lipids; i.e. phospholipids and cholesterol) was higher in group 2 (14.0 ± 2.3 g) than in group 1 (6.0 ± 0.9 g; $P<0.05$). The deficits of reserve lipids mass in tumour-bearing rats, expressed as a percentage of the body reserve lipid mass of controls, did not differ between the two groups (38.9 ± 4.9% in group 1 *vs* 40.1 ± 4.5% in group 2).

*Ashes* The difference of mass of carcass ashes between tumour-bearing and control rats was larger in group 1 than in group 2 ($P<0.05$, Figure 2A). The statistical significance was lost when the difference was expressed as percentage of carcass mass difference.

### *Tumour composition*

Tumour masses were not different between groups 1 and 2 (Table II). The composition of the tumour was similar in group 1 and in group 2 (water: 20.4 ± 1.3 *vs* 19.0 ± 1.0 g, protein: 3.4 ± 0.2 *vs* 3.1 ± 0.2 g, lipids: 0.31 ± 0.03 *vs*

0.23 ± 0.02 g, respectively). Tumoural lipids corresponded to 5.1 ± 0.5% of the deficit of body lipids in tumour-bearing rats in group 1, against 2.0 ± 0.4% in group 2 ($P<0.05$). Tumoural proteins corresponded to 28.6% ± 1.5% of the deficit of body proteins in group 1 against 21.3 ± 2.6% in group 2 ($P<0.05$).

### Discussion

The main results of this study are the following: (i) Despite the differences in body mass and initial body fuel reserves, mass and composition of the tumour were the same in young and adult rats after the same duration of tumour development. (ii) In contrast, adult tumour-bearing rats had a negative nitrogen balance, against a positive one in youngs. Since in adults protein accretion is reduced, this suggests that protein metabolism was more altered. (iii) Lipid deficit was higher in adult than in young tumour-bearing rats. (iv) Despite the difference in body mass, food intake was the same in tumour-bearing rats both ages.

In contrast to the present study, in which the rats still had some food intake, in the experiments by Sauer and Dauchy (1987) starvation accelerated tumour growth in adult but not in immature rats. These authors had ascribed this to the increased availability of circulating fat-derived nutrients from the host during starvation. That such an acceleration of tumour growth was absent in our conditions agrees with their interpretation. The capacity of the tumour to draw on the host lipid stores, rather than the rate of utilisation of the circulating nutrients might be a limiting factor for tumour growth.

Alterations in the host composition (Eden *et al.*, 1983; Lundholm, 1986), manifested by an increase in relative water content (Rechcigl *et al.*, 1961) and a decrease in body mass, in body protein and lipid (Brennan & Burt, 1981; Beck & Tisdale, 1991) have been described both in young and adult tumour-bearing rats and mice. Tumour requirements take priority over the demands of normal growth (Mider *et al.*, 1948). For adult rats, these results have been confirmed in the present study, in which tumour-bearing resulted in a loss in body mass. In young rats, however, in which growth rate is normally higher, host body mass still increased (Table II). This better conservation of body mass was confirmed by the nitrogen balance of young and adult rats (Table I). The balance of young rats still remained positive while that for adult rats became negative. This difference between adult and young rats is remarkably similar to previous observations of underfed rats, in which body mass decreased in adult rats whereas it still increased in young rats (Widdowson & McCance, 1956). Importantly, however, our study also indicates that the overall deficit in body mass of tumour-bearing rats, compared to control rats, was similar in young and adult rats. Although it was impaired, the young rats still maintained a high growth rate.

The deficit of protein mass in tumour-bearing rats was not different in young and adult rats, indicating that in absolute values the protein cost of the tumour for the host was the same in adult and young rats. Accordingly, since the young rats had a lower body protein content than adult rats, their protein deficit expressed as a fraction of total body proteins of controls was higher. However, young rats still maintained a positive nitrogen balance, contrary to the adults. A major difference between young and adult rats is that young rats have a much higher protein accretion. This, rather than body protein content, would be the key factor in the protein balance of tumour-bearing rats.

Although tumour metabolism is almost exclusively glycolytic, lipids are utilised by the host to provide carbohydrates to the tumour (Mulligan & Tisdale, 1991). Despite similar tumoural composition and mass in young and adult rats, the deficit of reserve lipids in tumour-bearing rats was higher in adult than in young rats. This suggests that the lipid cost of the tumour was higher in adults than in young rats. This difference of pattern of body reserves depletion parallels the difference of body composition alteration of underfed rats of various ages (Widdowson & McCance, 1956). Thus, the body composition alteration induced by cancer cachexia was attenuated in young rats, in agreement with a better metabolic efficiency in these rats as compared to adults.

Food intake was reduced both in young and adult tumour-bearing rats. Remarkably, it stabilised at a nearly identical level in both groups (Table I). Consequently, one would have expected a larger reduction in absolute terms in adults compared with controls than in youngs. This effect did not reach statistical significance, owing presumably to the wide dispersion of data. Moreover, although experimentally restricted controls may differ from spontaneously anorexic animals, controls pair-fed to tumour-bearing rats should be investigated in further studies.

In conclusion, body protein and lipid deficit due to sarcoma-bearing in rats varies with age. Most remarkably, presumably due to their better protein accretion, young rats unlike adult rats still maintained a positive nitrogen balance in spite of a higher relative protein deficit.

Dr Claude Leray and Ms Huguette Beekenkamp are gratefully acknowledged for their help with the dosage of lipids and ashes. We thank Dr Didier Attaix for providing the tumour. This work was supported by a grant from the Association pour la Recherche sur le Cancer.

### References

BECK, S.A. & TISDALE, M.J. (1991). Lipid mobilising factors specifically associated with cancer cachexia. *Br. J. Cancer*, **63**, 846–850.

BRENNAN, M.F. & BURT, M.E. (1981). Nitrogen metabolism in cancer patients. *Cancer Treat. Reports*, **65** (Suppl. 5), 67–78.

COSTA, G. (1977). Cachexia, the metabolic component of neoplastic diseases. *Cancer Res.*, **37**, 2327–2335.

EDEN, E., LINDMARK, L., KARLBERG, I. & LUNDHOLM, K. (1983). Role of whole body lipids and nitrogen as limiting factors for survival in tumor-bearing mice with anorexia and cachexia. *Cancer Res.*, **43**, 3707–3711.

FOLCH, J., LEES, M. & SLOANE-STANLEY, G.H. (1957). A simple method for the isolation and purification of total lipids from animal tissues. *J. Biol. Chem.*, **32**, 1570–1574.

GOODMAN, M.N., LARSEN, P.R., KAPLAN, M.M., AOKI, T.T., YOUNG, V.R. & RUDERMAN, N.B. (1980). Starvation in the rat. II. Effect of age and obesity on protein sparing and fuel metabolism. *Am. J. Physiol.*, **239**, E277–E286.

LUNDHOLM, K.G. (1986). Body composition changes in cancer patients. *Surg. Clin. North America*, **66**, 1013–1023.

LUNDHOLM, K., EDSTRÖM, S., KARLBERG, I., EKMAN, L. & SCHERSTEN, T. (1980). Relationship of food intake, body composition and tumor growth to host metabolism in non-growing mice with sarcoma. *Cancer Res.*, **40**, 2516–2522.

MIDER, G.B., TESLUK, H. & MORTON, J.J. (1948). Effects of Walker carcinoma 256 on food intake, body weight and nitrogen metabolism of growing rats. *Acta Union Intern. Contre Cancer*, **6**, 409–420.

MORRISON, S.D. (1983). *In vivo* estimation of size of experimental tumors. *J. Natl Cancer Inst.*, **71**, 407–408.

MULLIGAN, H.D. & TISDALE, M.J. (1991). Metabolic substrate utilization by tumour and host tissues in cancer cachexia. *Biochem. J.*, **277**, 321–326.

RAUSER, G., FLEISCHER, S. & YAMAMOTO, A. (1969). The two dimensional thin layer chromatographic separation of polar lipids and determination of phospholipids by phosphorus analysis of spots. *Lipids*, **5**, 494–496.

RECHCIGL, M., GRANTHAM, F. & GREENFIELD, R.E (1961). Studies on the cachexia of tumor-bearing animals. Body weight changes, carcass composition, and metabolic studies. *Cancer Res.*, **21**, 238–251.

SAUER, L.A. & DAUCHY, R.T. (1987). Blood nutrient concentrations and tumour growth *in vivo* in rats: relationships during the onset of an acute fast. *Cancer Res.*, **47**, 1065–1068.

SAUER, L.A., NAGEL, W.O., DAUCHY, R.T., MICELI, L.A. & AUSTIN, J.E. (1986). Stimulation of tumor growth in adults rats *in vivo* during an acute fast. *Cancer Res.*, **46**, 3469–3475.

TISDALE, M.J. (1991). Cancer cachexia. *Br. J. Cancer*, **63**, 337–342.

TRACEY, K.J. (1992). TNF and other cytokines in the metabolism of septic shock and cachexia. *Clin. Nutr.*, **11**, 1–11.

WARREN, S. (1932). The immediate causes of death in cancer. *Am. J. Med. Sci.*, **184**, 610–616.

WIDDOWSON, E.M. & MCCANCE, R.A. (1956). The effects of chronic undernutrition and of total starvation on growing and adult rats. *Br. J. Nutr.*, **10**, 763–773.